# A study on the material properties of novel PEGDA/gelatin hybrid hydrogels polymerized by electron beam irradiation

**DOI:** 10.3389/fchem.2022.1094981

**Published:** 2023-01-09

**Authors:** Tuğçe Şener Raman, Mathias Kuehnert, Olesya Daikos, Tom Scherzer, Catharina Krömmelbein, Stefan G. Mayr, Bernd Abel, Agnes Schulze

**Affiliations:** ^1^ Institute of Surface Engineering (IOM), Leipzig, Germany; ^2^ Wilhelm-Ostwald-Institute for Physical and Theoretical Chemistry, Institute of Chemical Technology of the University Leipzig, Leipzig, Germany

**Keywords:** hybrid hydrogel, electron beam, gelatin, PEGDA, mechanical properties of crosslinked polymers

## Abstract

Gelatin-based hydrogels are highly desirable biomaterials for use in wound dressing, drug delivery, and extracellular matrix components due to their biocompatibility and biodegradability. However, insufficient and uncontrollable mechanical properties and degradation are the major obstacles to their application in medical materials. Herein, we present a simple but efficient strategy for a novel hydrogel by incorporating the synthetic hydrogel monomer polyethylene glycol diacrylate (PEGDA, offering high mechanical stability) into a biological hydrogel compound (gelatin) to provide stable mechanical properties and biocompatibility at the resulting hybrid hydrogel. In the present work, PEGDA/gelatin hybrid hydrogels were prepared by electron irradiation as a reagent-free crosslinking technology and without using chemical crosslinkers, which carry the risk of releasing toxic byproducts into the material. The viscoelasticity, swelling behavior, thermal stability, and molecular structure of synthesized hybrid hydrogels of different compound ratios and irradiation doses were investigated. Compared with the pure gelatin hydrogel, 21/9 wt./wt. % PEGDA/gelatin hydrogels at 6 kGy exhibited approximately up to 1078% higher storage modulus than a pure gelatin hydrogel, and furthermore, it turned out that the mechanical stability increased with increasing irradiation dose. The chemical structure of the hybrid hydrogels was analyzed by Fourier-transform infrared (FTIR) spectroscopy, and it was confirmed that both compounds, PEGDA and gelatin, were equally present. Scanning electron microscopy images of the samples showed fracture patterns that confirmed the findings of viscoelasticity increasing with gelatin concentration. Infrared microspectroscopy images showed that gelatin and PEGDA polymer fractions were homogeneously mixed and a uniform hybrid material was obtained after electron beam synthesis. In short, this study demonstrates that both the presence of PEGDA improved the material properties of PEGDA/gelatin hybrid hydrogels and the resulting properties are fine-tuned by varying the irradiation dose and PEGDA/gelatin concentration.

## 1 Introduction

Hydrogels are three-dimensional polymeric networks made of crosslinked hydrophilic polymer chains that are able to absorb and retain a large amount of water and other biological fluids without dissolving (Hoffman, 2012; Cavallo et al., 2017). Because of their water content and adjustable physicochemical properties, they have become an increasingly preferred material in tissue engineering to mimic soft human tissues (Lutolf and Hubbell, 2005; Ramires et al., 2005; Palmese et al., 2019). Also, they can be applied in various areas, especially in biomedical applications, such as drug delivery systems (Dangi et al., 2022; Teja Surikutchi et al., 2022), contact lenses (Shin et al., 2020; Tran et al., 2022) and wound dressings (Ratner and Hofmann, 1976; Shao et al., 2023).

There are several kinds of preparation methods for hydrogels and basically, these can be divided into two main methods according to their mechanism of crosslinking: physical and chemical. (Rosiak and Ulanski, 1999; Akhtar et al., 2016; Garg and Garg, 2016). Physical crosslinking can be achieved *via* hydrophobic association, chain aggregation, crystallization, polymer stereo complexation, chain complexion, and hydrogen bonding while in chemically crosslinked gels, the presence of covalent bonds between different polymer chains prevents their dissolution in solvents. In comparison with physically cross-linked gels, chemical gels have a higher mechanical strength, stability, and longer degradation time (Ullah et al., 2015; Akhtar et al., 2016). Although most techniques employed to synthesize chemically cross-linked gels need initiators, electron beam irradiation does not require the addition of adding any initiator to the reaction mixture and this prevents the formation of unwanted products (Wang et al., 2007; Wang et al., 2007). Electron beam synthesis of the hydrogel results in a homogeneous structure of the hydrogel network, while radiation cross-linking is a well-established technique for developing sterile hydrogel wound dressings (Rosiak and Yoshii, 1999; Caló and Khutoryanskiy, 2015).

During the synthesis of the hydrogel, high-energy electrons break the bonds within the polymeric chains by homolytic cleavage, e.g. of C-H bonds, leading to the formation of radicals (Hennink and Nostrum, 2002). In addition, radiolysis of water initiates the formation of macro radicals, which participate in intra-and intermolecular recombination reactions with free radicals and eventually form a crosslinked polymer network with a stable and durable structure (Treloar, 2005).

Gelatin is derived from the hydrolysis of collagen which is the major protein component of bones, tendons, and skin, and hence, it is a remarkably alluring material in several areas of research. (Fu et al., 2000). Furthermore, it offers high biocompatibility and biodegradability, as it is unlikely to cause irritation or incompatibility in biological applications and degrades without releasing toxic byproducts (Wang et al., 2007; Wisotzki et al., 2014). However, the network structure of gelatin formed by hydrogen bonding is unstable. The network becomes destroyed as the temperature rises above the gel temperature, resulting in poor thermal and low mechanical properties (Cui et al., 2022). Moreover, materials made of gelatin in the dry state show brittleness, low flexibility, and extremely fast degradation rate problems (Chang et al., 1998; Tan et al., 2009; Gorgieva and Kokol, 2011). To overcome this problem, the natural polymer gelatin is blended with a synthetic polymer to enhance the mechanical characteristics (Yung et al., 2007). Polyethylene glycol (PEG)-based hydrogels have been more and more explored in the last decades for tissue engineering and drug delivery applications, due to their convenient non-fouling character and adjustable crosslinking density (Hern and Hubbell, 1988; Cascone, Sim, and Dowries, 1995; Bryant and Anseth, 2002; Broderick et al., 2005; Engberg and Frank, 2011). Especially, acrylated or methacrylated PEG derivatives can also be cross linked into hydrogels by gamma irradiation to obtain hydrogels that may have complex shapes and microstructures, crosslinking density gradients, and may be polymerized directly (Nemir, Hayenga, and West, 2010). Also, PEG hydrogels are widely used because their mechanical properties can be easily tailored (Tong and Yang, 2014).

In this study, by combination of PEGDA and gelatin, PEGDA/gelatin hybrid hydrogels are synthesized bei electron beam treatment and are expected to have high mechanical properties and biocompatibility, as well as promising biodegradability. Moreover, pure gelatin scaffolds are too fragile to be used in practice. For this reason, the combination of PEGDA with gelatin will significantly increase flexibility. For this purpose, the ratio of gelatin and PEGDA and the dose of electron irradiation were varied. The cross linked network of PEGDA/gelatin hybrid hydrogels was investigated by using infrared spectroscopy. The thermo-mechanical properties, swelling behavior, gel content, and morphology of the resulting hydrogels were also studied. The physical properties were characterized to identify the optimal ratio of synthetic and biopolymer and irradiation dose for use as a hydrogel wound dressing.

## 2 Materials and methods

### 2.1 Materials

Type A gelatin from porcine skin with a high molecular weight and Bloom number of 300, polyethylene glycol diacrylate (PEGDA, Mn = 700 g mol^−1^), phosphate buffered saline solution (PBS; pH = 7.4) were obtained from Sigma-Aldrich (Saint-Louis, MO, United States). The chemicals were used as received without additional purification.

### 2.2 Preparation of hydrogels

Desired concentrations in phosphate-buffered saline (PBS) solution were prepared, as indicated in Table 1. Gelatin powder was allowed to swell in the solution for 30 min before the solution was heated to 60 C and stirred until a transparent solution was obtained. Approximately 1 mL of the solution was pipetted into 35 mm polystyrene petri dishes. The formulation was polymerized using a 10 MeV linear accelerator (MB10-30 MP, Mevex Corp, Stittville, ON, Canada). Differential doses were irradiated to investigate the influence of the crosslinking procedure on the resulting hydrogels. These doses were used: 1 × 3°kGy (one-time 3 kGy), 2 × 3°kGy, 3 × 3°kGy, 4 × 3°kGy, 5 × 3°kGy, and 1 × 6°kGy using the P26G4 hybrid hydrogel. The irradiation duration was 1.05 s and 2.1 s for the samples with 3 kGy and 6 kGy differential doses, respectively. To prevent heating during irradiation, the samples were cooled by an air draft. Afterwards, the hydrogels were washed twice in PBS solution and twice in milli-Q water for a total of 4 h and dried for 24 h at 40 C. The resulting gels had a thickness of 1 mm.

**TABLE 1:** Precursor formulations.

Formulation	P0G6	P30G0	P29G1	P28G2	P27G3	P26G4	P25G5	P24G6	P23G7	P22G8	P21G9
PEGDA (wt%)	0	30	29	28	27	26	25	24	23	22	21
Gelatin Type A (wt%)	6	0	1	2	3	4	5	6	7	8	9

While determining these composite gradients with a low electron beam in Table 1, the elastic modulus of the skin was considered as a target value. This value is approximately 1 MPa (Balakrishnan et al., 2022). In the study, first of all, the mechanical properties of pure gelatin and PEGDA were determined, and then PEGDA/gelatin compounds were investigated. PEGDA/gelatin mixtures were selected, which had a value comparable to the elastic modulus of the skin.

### 2.3 Rheology

A MCR300 rheometer (Anton Paar, Graz, Austria) was used to determine the storage modulus and the loss factor of the hybrid hydrogels at 25°C. The rheometer was used with a 10 mm probe head. A metal punch was used to cut hybrid hydrogel circles of 10 mm in diameter. The probe head was pressed on the sample with 10 N. All values were recorded at 1 Hz.

### 2.4 Tensile test

The tensile strength test was measured using a 3 kN Inspect mini (Hegewald & Peschke GmbH, Nossen, Germany) strength testing machine. The test speed was set to 1 mm/min in the Labmaster software (Version 2.8.12.12) supplied with the Inspect mini.

The tensile strength R_m_ was calculated by the machine software from the formula:

$$R_{m}=\frac{F_{m}}{\bar{a}\;\bar{b}}$$ (1)

where: F_m_ maximum strength, $\bar{a}$ average measured sample thickness, $\bar{b}$ average measured sample width. Each sample’s average width and thickness were entered separately into Labmaster.

### 2.5 Swelling ratio and gel fraction

The effect of concentration and electron irradiation on the swelling ability of gelatin was determined by storing samples in pure water and monitoring their weight until equilibrium was reached (approximately 24 h) at room temperature. The dried samples were placed at different time intervals in distilled water to swell. The swelling measurement was determined after taking out the soaked samples from distilled water, weighing them as soon as the surface droplets were wiped off with wet paper. The samples were then placed back into distilled water, and the protocol was repeated until no further weight change was observed.

The swelling ratio (q) was determined using the following equation (Bal et al., 2014):

$$q=\frac{m_{wet}}{m_{dry}}x100$$ (2)

where m_wet_ is the mass of the wet hybrid hydrogel after swelling and m_dry_ is the mass of the dry hybrid hydrogel. For the gel fraction step, after the dry hydrogels were soaked in distilled water for 24 h, they are dried for 24 h at 40°C. The gel fraction “Gel” is the insoluble part of the hybrid hydrogel. The gel fraction ratio was used in the following equation (Zhu, 2010):

$$Gel=\frac{W}{W_{o}}$$ (3)

where W_o_ is the initial weight of the sample; W is the weight of the dry insoluble part.

### 2.6 Thermal analysis

Thermal stability tests were performed by thermogravimetric analysis and a Pyris 1 TGA (Perkin Elmer, Waltham, MA, United States) was employed. The temperature range was between 20°C and 800 C. The heating rate was 10°C min^−1^, and air was used as a purge gas. Moreover, differential scanning calorimetry (DSC) was used to measure the glass transition temperature (Tg) of the hydrogels.

### 2.7 FTIR

Attenuated total reflectance-infrared spectroscopy (ATR-FTIR) was measured to evaluate the chemical composition of the hybrid hydrogels using a Bruker Tensor II Fourier trans-form infrared (FTIR) spectrometer from Bruker Optik GmbH, Ettlingen, Germany. Spectra were recorded in the range of 4,000–750 cm^−1^ for each sample. Various concentrations of dry hybrid hydrogels and several doses from 1 × 3 to 5 × 3 kGy using P26G4 hybrid hydrogels were investigated.

### 2.8 Scanning electron microscopy

Dried P29G1, P26G4, and P21G9 samples were imaged in the scanning electron microscope using an Ultra-55 microscope equipped with a Gemini Detector (both from Zeiss, Jena, Germany). For this, the samples were fractured to achieve a cross-sectional surface. The fractured cross-sections were placed facing upwards. After then, these fractured cross-sections were investigated.

### 2.9 Infrared microspectroscopy

Infrared spectral imaging was carried out with a Hyperion 3,000 IR microscope coupled to a Tensor II FTIR spectrometer (Bruker Optik GmbH, Ettlingen, Germany). The microscope is equipped with an ATR objective (provided with a Germanium crystal; n_D_ = 4.0) as well as with a 64 × 64 pixel focal plane array (FPA) detector. Spectral images of the hydrogel samples were recorded in the ATR mode. Each image covers a size of 32 × 32 μm. Taking into account the data of the ATR objective and the refractive index of PEGDA (n_D_ = 1.47; the index of gelatin is roughly the same: n_D_ = 1.5), the penetration depth of the probe light into the sample is about 760 nm at 1,093 cm^−1^, i.e., at the position of the most intense band in the spectrum of PEGDA corresponding to the asymmetric C-O-C stretching vibration. For an adequate signal-to-noise ratio, 128 accumulations were recorded for each image. The spectral resolution was set to 8 cm^−1^. For each hydrogel sample, several images were taken from various positions at both the top and the bottom side.

### 2.10 Determination of crosslinking density

The effect of changing PEGDA and gelatin concentrations on crosslinking density in the hybrid hydrogels was investigated. Meanwhile, the crosslinking density of the hybrid hydrogel synthesized by electron beam irradiation at various doses was determined. The theory of rubber elasticity assumes that gelatin is a flexible polymer that responds to thermal energy like an ideal elastic material. On this basis, the entropy-driven relationship is comparable to that of an ideal gas. Using the theory of rubber elasticity, the crosslinking density of the hybrid hydrogels was calculated according to the following formula (Ilavský, 1993):

$$G^{\prime }=v_{c}RT$$ (4)

where the degree of crosslinking v_c_, G' is the storage modulus determined by rheology, R is the universal gas constant (8.314 J mol^−1^ K^−1^), and T is the temperature during the measurement (298 K).

## 3 Results and discussion

### 3.1 Mechanical properties

**FIGURE 1:**
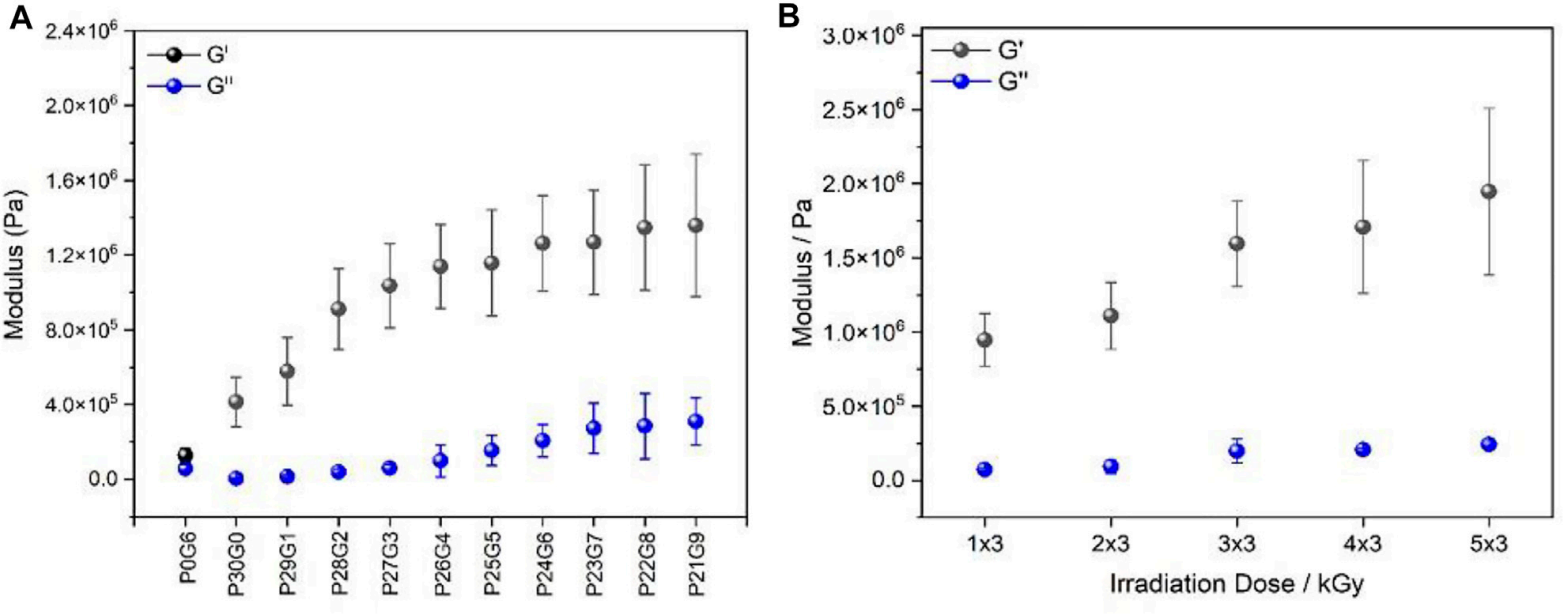
Rheological characteristics: storage modulus G′ and loss modulus G′′ of **(A)** different PEGDA/gelatin ratios of hybrid hydrogels irradiated with 6 kGy and **(B)** P26G4 hydrogel irradiated with different electron irradiation doses. Moduli were measured at a frequency of 1 Hz. Error bars indicate the standard deviation.

Rheological measurements on the PEGDA/gelatin hybrid hydrogels with variation in component ratio indicated a clear ability to control their viscoelastic properties. For this, the rheological properties of hydrogels, such as storage modulus G' and loss modulus G'' in the linear viscoelastic region, were determined in order to characterize the crosslinking degree. By increasing the gelatin proportion and decreasing PEGDA proportion in the corresponding hybrid hydrogels irradiated with 6 kGy, rheometry measurements displayed a significant increase in the storage modulus of the samples as shown in Figure 1A while pure gelatin had a lower storage modulus with 0.13 MPa, P21G9 had the highest storage modulus with 1.40 MPa P21G9 achieved an elasticity gain of 1,078% compared to the pure gelatin hydrogel. Also, it is clearly visible that the storage modulus of the samples increased with increasing irradiation doses in Figure 1B. The storage modulus of P26G4 was increased by 205% from 1 × 3 kGy to 5 × 3 kGy.

The crosslinking densities of all hydrogels were calculated from rubber elasticity theory using the G' value and Eq. 4. The values are displayed in Table 2. It was found that increasing the irradiation dose enhanced the formation of radicals in the reaction mixture, so that stronger crosslinking occurred, and a high storage modulus was obtained (Nasef et al., 2019). The crosslinking density of the different compound ratios of the corresponding hybrid hydrogels irradiated with 6 kGy was in a range of 167.4 mmol L^−1^ and 543.5 mmol L^−1^ while pure gelatin as well as pure PEGDA possessed the lowest crosslinking density (23.3 mmol L^−1^ and 51.8 mmol L^−1^, respectively). Moreover, the P26G4 hybrid hydrogel irradiated with 5 × 3 kGy had the highest crosslinking density with 785.6 mmol L^−1^. The crosslinking density of the P26G4 hybrid hydrogel was increased by 205% by irradiation with 5 × 3 kGy compared to 5 × 3 kGy. Obviously, the crosslinking density reached the highest value for hydrogels prepared with increasing gelatin concentration and high irradiation dose.

**TABLE 2:** Crosslinking density values calculated from the dynamic moduli.

Formulation (1 × 6 kGy)	P0G6	P30G0	P29G1	P28G2	P27G3	P26G4	P25G5	P24G6	P23G7	P22G8	P21G9
*G* ^*’*^ */MPa*	0.13	0.41	0.58	0.91	1.09	1.14	1.16	1.26	1.27	1.35	1.40
n */mmol · L* ^*−1*^	51.8	167.4	233.6	367.6	441.5	459.8	467.2	509.6	526.4	543.5	566.6
tan*(δ)*	0.287	0.013	0.027	0.044	0.057	0.084	0.137	0.161	0.219	0.215	0.217
*Formulation (P26G4)*	1 × 3 kGy		2 × 3 kGy		3 × 3 kGy		4 × 3 kGy		5 × 3 kGy		
*G* ^*’*^ */MPa*	0.95		1.11		1.59		1.71		1.95		
n */mmol · L* ^*−1*^	382.8		447.6		644.2		689.1		785.6		
$\tan\left(\delta \right)$	0.080		0.080		0.120		0.130		0.133		

Finally, the loss factor tan(δ), which is the ratio of loss modulus to storage modulus, can be used to characterize a material’s viscoelastic properties (Table 2). The more pronounced the elastic properties are, the lower the value of tan(δ) (Jiang et al., 1999), i.e., tan(delta)<<1. It is shown that irradiation dose and increased gelatin proportion affected the viscoelastic properties of the respective hydrogels. The results reveal that increased gelatin concentration and irradiation doses had a significant influence on the mechanical properties of the resulting hydrogels.

**FIGURE 2:**
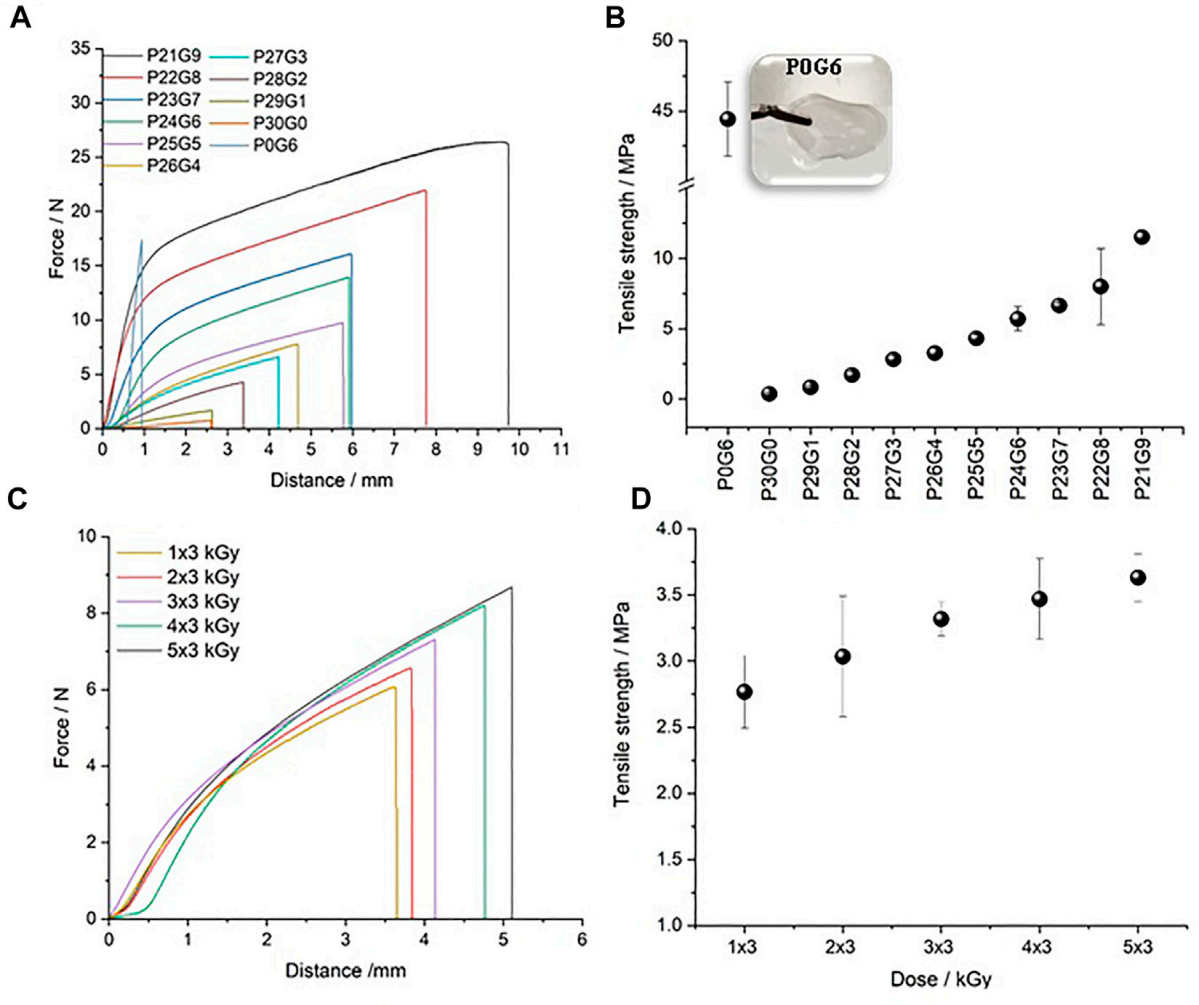
Tensile test and tensile strength: **(A,B)** different PEGDA/gelatin ratios of hybrid hydrogels irradiated with 6 kGy and **(C,D)** P26G4 hydrogel irradiated with different electron irradiation doses. Error bars indicate the standard deviation.

Rheological characterization is very significant to know the viscoelastic nature of the hydrogel and the gel strength in a shearing action. The frequency-dependent properties of the hydrogels showed a prominent difference between the storage (elastic component) and loss modulus (viscous component) which is obvious proof of the elastomeric nature of the hydrogel. Storage modulus increases with increasing gelatin ratio and irradiation dose. This can be attributed to the elastic dominance property of the hydrogel without any phase separation. Moreover, it helps to understand the crosslinking density of hydrogels. On the other hand, to further investigate the effects of PEGDA/gelatin concentration and irradiation dose on the mechanical properties of the hydrogels, tensile tests were performed (Figure 2). The obtained tensile strength is used to determine the mechanical strength of the hydrogel. A larger value indicates higher stability of the hydrogel (Li et al., 2020). Compared to hybrid hydrogels with different PEGDA/gelatin ratios in Figure 2A, pure gelatin (P0G6) has the lowest breaking point which shows that it has a brittle structure whereas the breaking point increases with the gelatin ratio of hybrid hydrogels. Figure 2B indicates that as the content of gelatin increases, the tensile strength of the hydrogel also increases. The higher strength of hydrogels may be due to their high degree of polymerization (Xiang et al., 2022). Thus, also tensile test results confirms the beneficial effect on the mechanical properties by combining gelatine and PEGDA in a cross linked hydrogel.

Figures 2C, D presents the mechanical strength of the P26G4 hydrogel irradiated with different electron irradiation doses. The breaking point and tensile strength rise slightly with increasing irradiation dose. An increase in both modulus (G’ and G*) and tensile strength with increasing PEGDA/gelatin ratio and irradiation dose indicate an increased crosslinking density of the hydrogel structure (Sai Pavan Grandhi et al., 2018). An increase in tensile strength is seen to positively correlate with an increase in modulus (measured by rheometer) indicating the complementary nature of measurements by these instruments in determining hydrogel properties.

### 3.2 Swelling ratio and gel fraction

**FIGURE 3:**
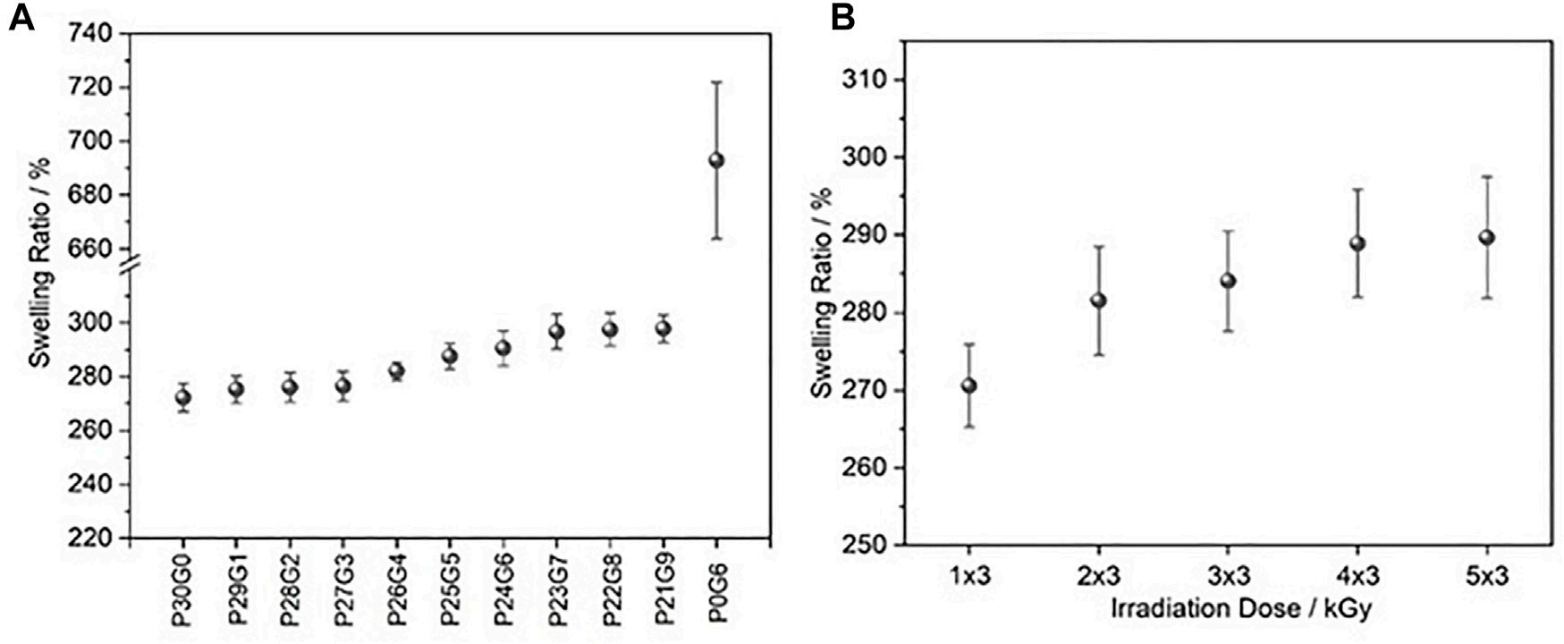
The swelling ratio of hydrogels after immersion in water for 24 h **(A)** at different PEGDA/gelatin ratios and electron doses of 6 kGy and **(B)** with fixed PEGDA/gelatin ratio of P26G4 after polymerization with different irradiation doses. Error bars indicate standard deviations.

The hydrogel’s capacity to absorb water allows cells to thrive in a nutrient and water-rich environment, which aids wound healing (Hajighasem and Kabiri, 2013). The swelling ratio of different hydrogels with variations of PEGDA/gelatin ratios and irradiation doses was investigated by measuring the mass difference of the hydrogels before and after immersion in distilled water. All nine variations of hybrid hydrogels exhibited high water content (Figure 3A). It can be noted that P21G9 had the maximum swelling ratio (around 297.76%), while P30G0 (pure PEGDA) had the minimum swelling ratio (approximately 272.16%). P0G6 (pure gelatin) had an even higher swelling ratio than P21G9 with 692.89% which was expected since this formulation has the lowest total polymer concentration. For the mixed samples, the water uptake of the hydrogels increased the higher the gelatin content, and the higher the gelatin network content resulted in greater water uptake due to the higher dry gelatin content available for water uptake (Wisotzki et al., 2014). Swelling is the first step in polymer dissolution and is created with the interaction between liquid molecules and the polymeric network. After this step, the solvation of polymer chains usually follows. Due to chemically bonded hydrocarbon chains of cross-linked polymers, they don’t dissolve even when immersed in a solvent. The studies of Riedel et al. (2017) demonstrated that gelatin can be cross linked using an electron beam, which represents a non-toxic crosslinking method, and the swelling properties of gelatin can be tuned without dissolving. The degree of polymeric network expansion is associated with the chemical character of solvent molecules and the pore structure of the polymer. Also, solvent uptake can be accepted as the sum of the processes swelling and filling of the pores (Sienkiewicz et al., 2017). In case there are pores in the hydrogel, overall solvent uptake might be higher. It could be created also pores with increasing doses which will contribute to the overall solvent uptake in swelling experiments. The studies of Demeter et al. (2022) present that collagen/PVP/PEO hydrogels synthesized with various irradiation doses show significant changes in their swelling properties as a function of the increased dose, while the water absorption capacity of hydrogels with dense networks is influenced by the ionic properties of the hydrogels. The swelling ratio of the here investigated hybrid hydrogels polymerized with different irradiation doses rose slightly with increased electron doses, as shown in Figure 3B.

**FIGURE 4:**
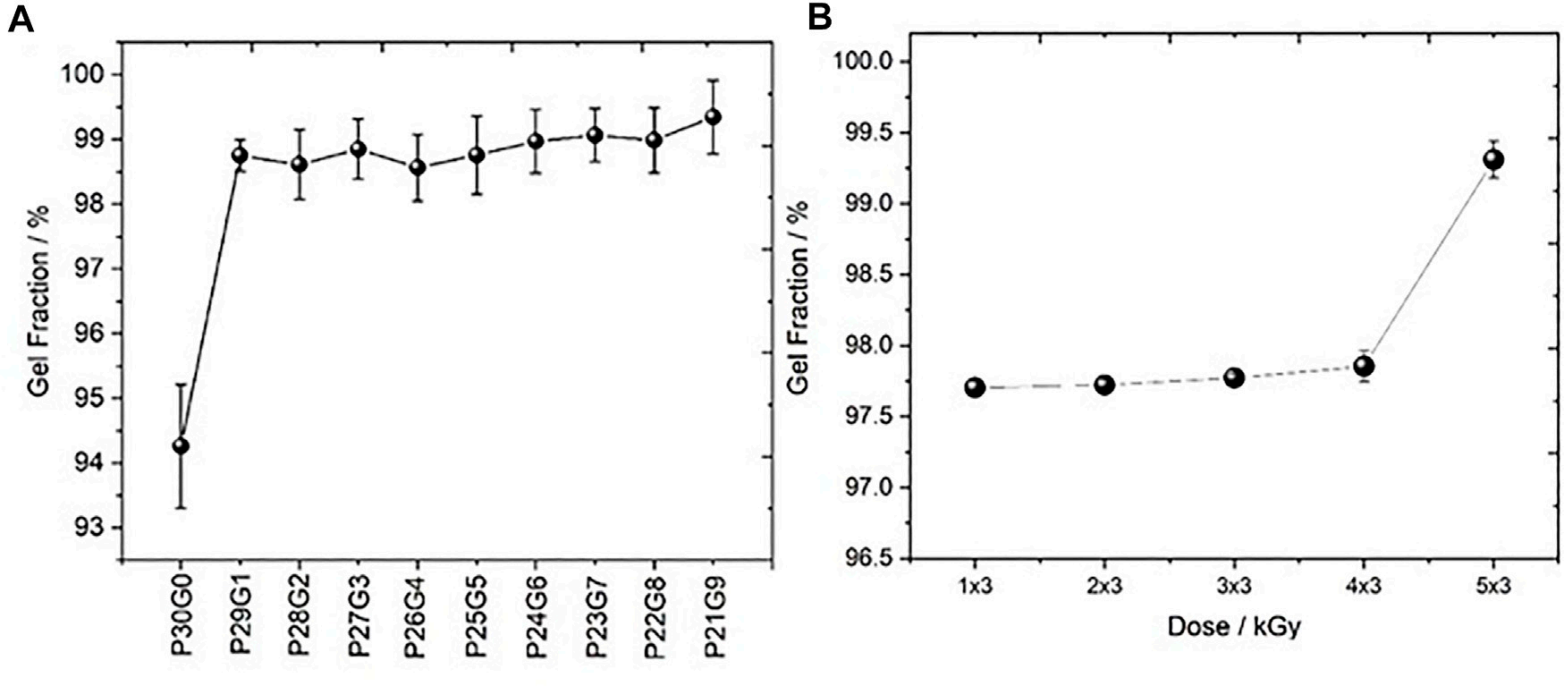
Gel fraction ratio of hydrogels **(A)** at different PEGDA/gelatin ratios and electron doses of 6 kGy and **(B)** with fixed PEGDA/gelatin ratio of P26G4 after polymerization with different irradiation doses. Error bars indicate standard deviations.

Gel fraction analysis was performed to determine the crosslinked fraction of the polymer in the hydrogel using Eq. 3. It was found that the gel fraction of the hydrogels increased along with increasing proportion of gelatin and irradiation dose as shown in Figure 4. It appears that the use of gelatin led to the generation of stronger cross linked networks. According to Cataldo et al. (2018) hybrid hydrogels had a higher gel fraction in the presence of gelatin than those without gelatin in the photopolymerized system. Both, the studies of Glass et al. (2019) and Cataldo et al. indicated that at higher radiation doses the crosslinking density of hydrogels is higher.

### 3.3 Thermal stability

**FIGURE 5:**
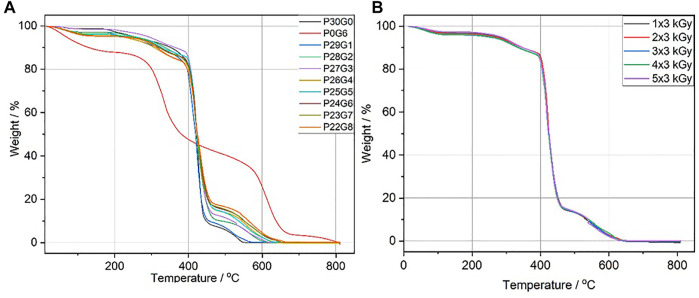
Thermal stability of hydrogels **(A)** at different PEGDA/gelatin ratios and fixed electron dose of 6 kGy **(B)** P26G4 hydrogel irradiated with different electron irradiation doses.

The thermal stability of the hydrogels is crucial for sterilization *via* autoclaving when used in biomedical applications. Autoclaving is a widely used method for sterilizing medical devices and it is usually performed at a temperature of at least 120 C (Galante, et al., 2018). In the study by Beard et al. (2021), Poloxamer 407 hydrogels were initially sterilized in order to be used as a drug delivery vehicle. Although the hydrogels are sterile after being synthesized by electron beam, sterilization is required before they can be reused in various biomedical applications. Autoclaving is an effective and useful method for this purpose. In order to determine the stability of the hydrogels after autoclaving, the selected hydrogels were autoclaved at 121°C for 20°min. After autoclaving, the samples were investigated regarding their mechanical stability with a rheometer. In Supplementary Figure S1, the rheological properties of hydrogels were compared before and after autoclaving; while pure gelatin was completely dissolved and remained unstable, hybrid hydrogels remained stable, and their mechanical properties didn’t change significantly. Moreover, the appropriate thermal stability of the hydrogels also provides a massive advantage for possible application. The results of the thermal stability analysis are shown in Figure 5. All samples were dried and stored in the air before conducting the experiments. All samples were dried and stored in air before conducting the experiments. Whereas hybrid hydrogels and P30G0 (pure PEGDA) had a small weight loss of 2%–5% at 100°C, P0G6 (pure gelatin) had a weight loss of 10%–12% at the same temperature in Figure 5A. This loss was the amount of water left in the samples because of the ambient humidity. The hydrogels were stable up to approximately 202°C. Since autoclaving is performed at lower temperatures, hydrogel sterilization at 120°C–134°C is ensured. The hydrogels started to degrade at temperatures higher than 202°C. The degradation took place in several steps. While the degradation of hybrid hydrogels was approximately 18%, it was 52% for pure gelatin at 400°C. The progress of hybrid hydrogel degradation increased with increasing gelatin proportion. In addition, it was found that the degradation of pure gelatin was almost advanced after 400°C. The degradation of hydrogels that were polymerized with different irradiation doses was similar as shown in Figure 5B.

### 3.4 FTIR

**FIGURE 6:**
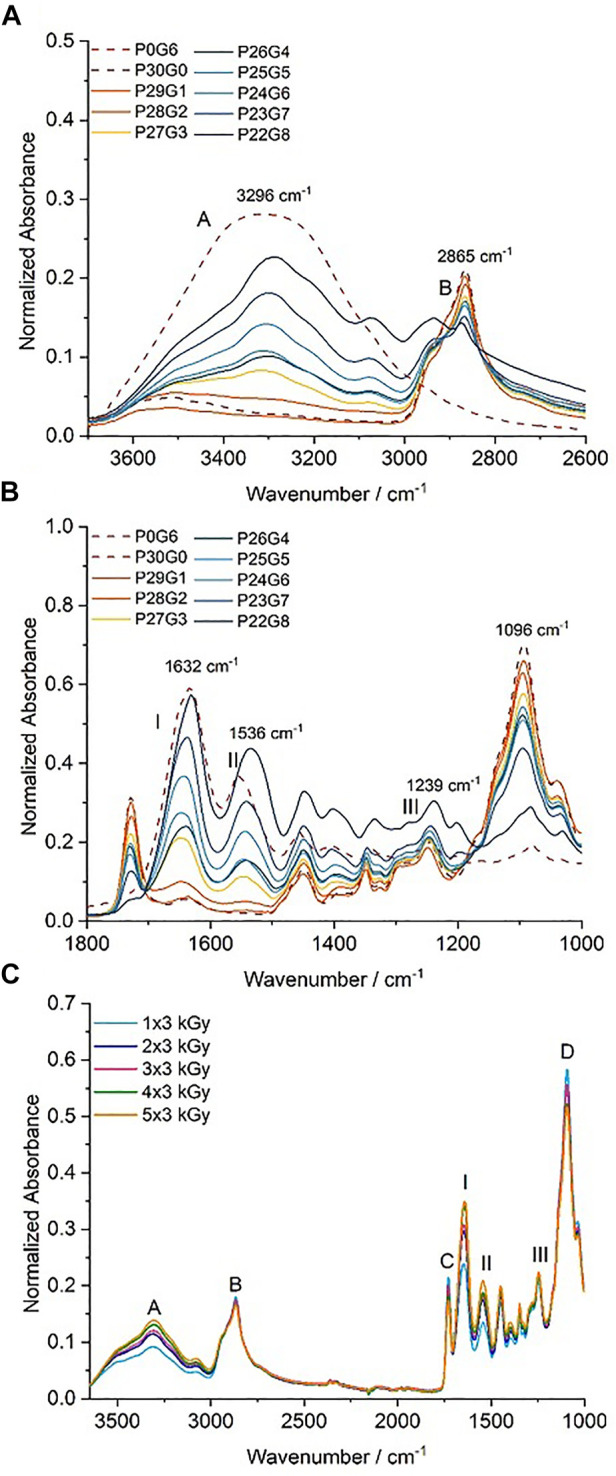
Attenuated total reflectance-infrared spectroscopy (ATR-FTIR) spectra of hydrogels with variation of **(A)** and **(B)** different PEGDA/gelatin proportions irradiated with 6 kGy, and **(C)** the differential irradiation doses with fixed PEGDA/gelatin concentration of P26G4.

The crosslinked network of PEGDA/gelatin hybrid hydrogels at various proportions and irradiation doses was investigated by means of ATR-FTIR spectroscopy. The analyses were performed on dried samples, that had been previously washed for 4 h to remove the unreacted soluble fraction. The ATR-FTIR spectra of the hydrogels are displayed in Figure 6. These results demonstrate that the signals of both gelatin and PEGDA were involved in the structure of PEGDA/gelatin hybrid hydrogels. Moreover, it was observed that the structure of the hybrid hydrogels undergoes some modifications with changing proportions of the two polymer compounds as well as electron irradiation dose. The spectrum of P0G6 (pure gelatin) exhibited an absorption band at 3,296 cm^−1^, corresponding to the stretching mode of N-H groups in Figure 6A. Whereas a broad absorption band around 3,300 cm^−1^ in the PEGDA/gelatin hybrid hydrogels spectra is associated with N-H bond stretching of the A band in gelatin (Shen et al., 2021), the peaks at 1,632, 1,554, and 1,242 cm^−1^ corresponded to the amide I (C=O stretching vibration), amide II (CN stretching/NH bending modes), and amide III bands (CN stretching/NH bending modes) of the gelatin, respectively (Elliott and Ambrose, 1950; Krimm and Bandekar, 1986; Mark 1999; Cosola et al., 2019). Higher wavenumbers for this band indicate a more helical structure, typically at 1,550 cm^−1^ for pure collagen (Kamińska and Sionkowska, 1996). While initially centered at 1,543 cm^−1^ for P29G1 hydrogels, the band clearly shifts towards a lower wavenumber and begins to reach a peak at 1,536 cm^−1^ for P22G8 hydrogels in Figure 6B. The shift towards a lower center point indicates that the gelatin structure is transitioning to an increasingly random coil structure. This shift can also be used to interpret the water loss during crosslinking and sample contraction (Riedel et al., 2019). The spectrum of pure PEGDA in hydrogels showed a C–H band stretching vibration at 2,865 cm^−1^, C=O stretching vibration at 1727 cm^−1^, and C–O stretching vibration at 1,094 cm^−1^, respectively (Miyazawa et al., 1956; Li et al., 2016). As illustrated in Figures 6A, B, where amide bands of gelatin are clearly visible in the spectra of hydrogels prepared with increasing gelatin proportion, absorption bands of PEGDA in hydrogels also rose with increased concentration of PEGDA. Generally, the absorption peaks at ∼3,290 and 1,550 cm^−1^ correspond to a gelatin triple-helix structure, and their positional shift and intensity decrease indicate the decay of such structures (Wisotzki et al., 2014). On the other hand, crosslinking resulted in an intensity increase of these bands, possibly due to the promotion of the formation of a larger number of triple helices) (Ishak et al., 2018). The obtained spectra (Figure 6C) of the P26G4 hybrid hydrogel polymerized with different irradiation doses showed that the absorption peaks around 3,290 cm^−1^ and 1,627 cm^−1^ also increased in intensity as a result of crosslinking, which was attributed to the enhanced organization of the helical configuration.

### 3.5 SEM investigation

**FIGURE 7:**
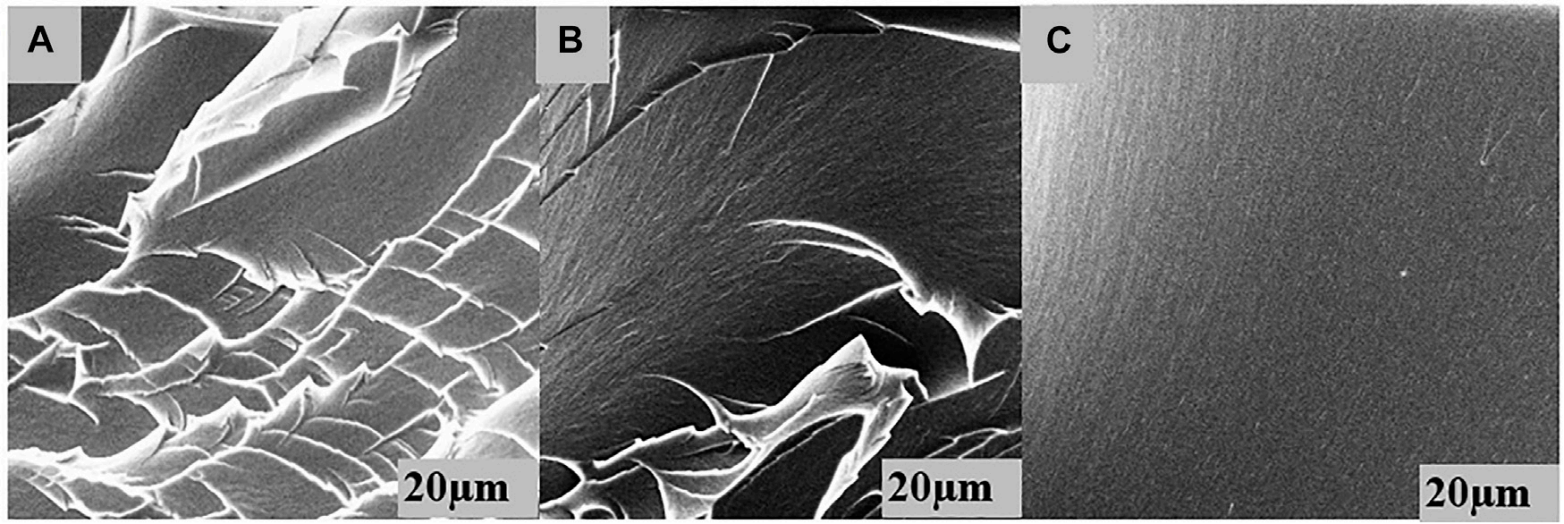
A cross**-**section view of the microstructures visible on fractured surfaces of dried PEGDA/gelatin hydrogel samples of **(A)** P29G1, **(B)** P26G4 and **(C)** P21G9 at 6 kGy.

SEM images were prepared from dried samples. Figure 7 displays cross-section view images of three different hybrid hydrogel compositions. Comparing the microscopic images of the hydrogels, a noticeably flaky matrix morphology was observed in the matrix of P29G1 hydrogels while P21G9 hydrogels exhibited a smooth surface. The increased gelatin and decreased PEGDA proportion in hydrogels caused the matrix surface to become smoother. Inner layers and irregularities in the cross-sections of the enhanced hydrogels may be related to increased rigidity and stiffness. It indicated a change to increasingly rigid structures in the hydrogel, as was observed in previous measurements with respect to increasing crosslinking degree (Ishak et al., 2018). In that study, it was suggested that the crosslinking agents caused more rigid structures of hydrogels.

### 3.6 Infrared microspectroscopy

**FIGURE 8:**
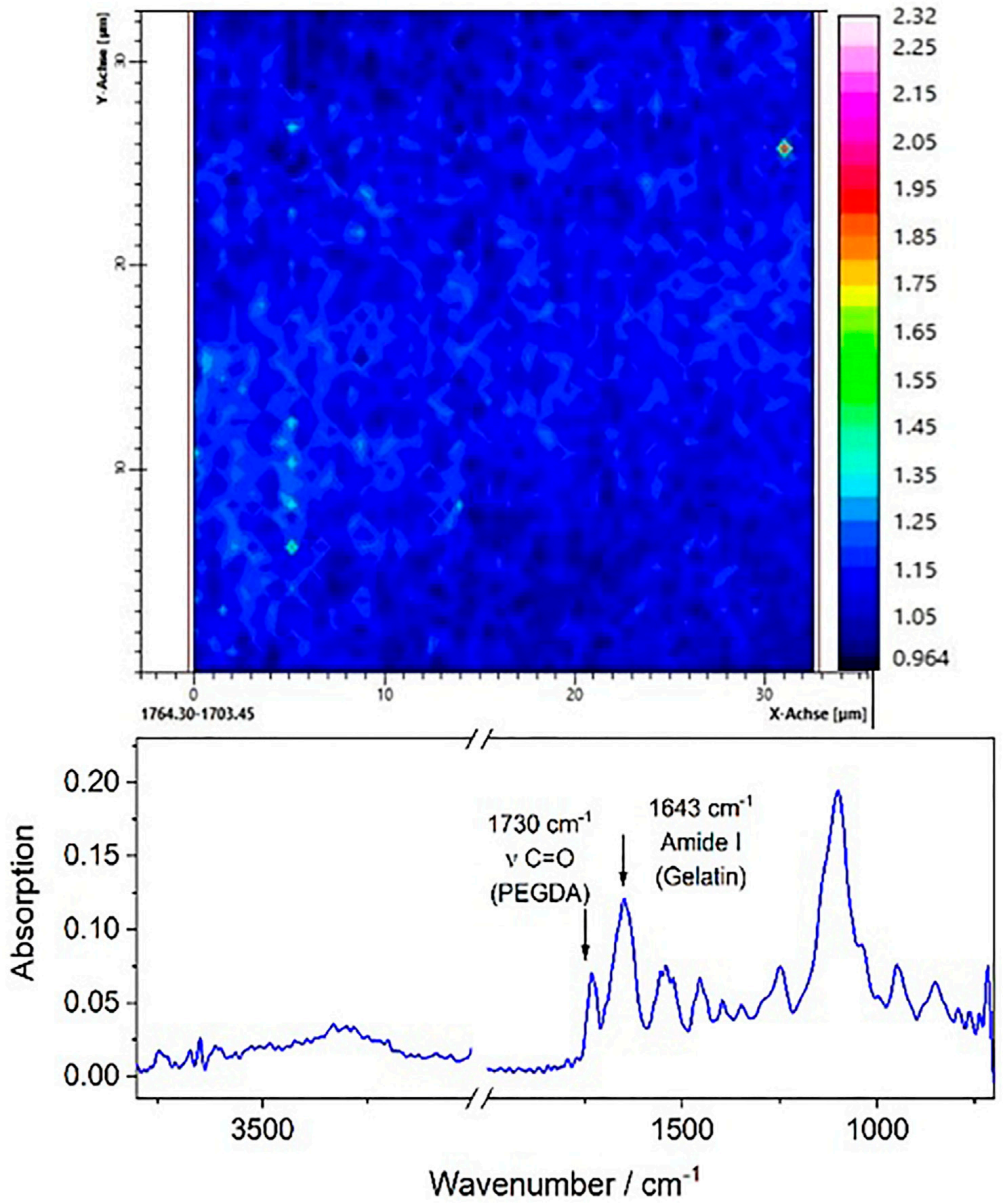
Infrared specrtal image of P26G4 hydrogels irradiated with 6 kGy. The bottom figure shows a represenative IR spectrum with characteristic bands of PEDGA and gelatin, respectively.

IR microspectroscopy carried out with an imaging detector offers the possibility to study the spatial homogeneity of samples. Figure 8 shows a spectral image of a P26G4 hydrogel sample highlighting the spatial distribution of the intensity of the carbonyl band in PEGDA. It is obvious that the intensity is roughly constant across the imaged surface area (32 × 32 μm) indicating the formation of a highly homogeneous polymer network without any phase separation or other inhomogeneities. Similar results were also obtained for the amide I band in gelatin. Spectral images were also taken at various positions of the upper and lower surface of the sample, which confirmed the high homogeneity. In the lower part of the figure, a characteristic spectrum of the hydrogel at a randomly selected point of the image is shown. The two bands, which are specific for gelatin and PEGDA, respectively, and which were used for establishing the images, are marked and assigned to the corresponding vibrations.

## 4 Conclusion

The material properties of PEGDA/gelatin hybrid hydrogels demonstrated the ability to be precisely controlled by irradiation dose and PEGDA/gelatin ratio. Rheometer measurements showed that PEGDA/gelatin hybrid hydrogels with increasing gelatin proportion were strengthened up to 1,078% compared to the pure gelatin hydrogel. Also, mechanical property in the strength of PEGDA/gelatin hydrogels was up to 205% with rising irradiation doses from 1 × 3 kGy to 5 × 3 kGy whereas the crosslinking density of hybrid hydrogels was increased at the same time for 205%. The swelling ratio of the corresponding hybrid hydrogels was effectively modified with respect to PEGDA/gelatin concentration and irradiation dose to achieve a maximum of 298%. The swelling ratio of PEGDA/gelatin hybrid hydrogels was increased with gelatin concentration since pure gelatin hydrogel showed a high swelling ratio. Additionally, it was determined that the swelling ratio of hydrogels declined with decreasing irradiation dose. On the other hand, the gel fraction ratios revealed that 97%–99% of the polymer network was successfully cross linked by electron irradiation. The synthesis of hybrid hydrogels by electron beam irradiation provides an option for application in drug delivery by reswelling with solutions including bioactive materials. In addition, hybrid hydrogels remain stable up to 202°C and this result enables hybrid hydrogels to be used in medical products with the possibility of autoclaving. Moreover, evaluation of FTIR-ATR measurements revealed that no significant alterations of the PEGDA/gelatin hydrogel backbone configuration during electron beam treatment, especially from 1 × 3 kGy to 5 × 3 Gy were observed. SEM images demonstrated that the structure of hydrogels has rigid and smooth fractured surfaces with increased gelatin concentration. Moreover, electron beam cured hybrid hydrogels were confirmed to be homogeneously networks as investigated by MIR-ATR spectral imaging. In summary, this study demonstrated the impact of both directed electron irradiation dose and variation of the PEGDA/gelatin proportion to achieve precise mechanical properties of hybrid hydrogels, including viscoelasticity, swelling properties, gel fraction, and structural configuration. Future work will focus on synthesizing PEGDA/gelatin hybrid hydrogels with antibacterial properties using antibacterial agents that play a major role in biomaterials.

## Data Availability

The original contributions presented in the study are included in the article/Supplementary Material, further inquiries can be directed to the corresponding author.
