# Supplementary material for: A study on the material properties of novel PEGDA/gelatin hybrid hydrogels polymerized by electron beam irradiation
Source: Front Chem. 2023 Jan 9;10:1094981. doi: 10.3389/fchem.2022.1094981 (PMC9868307; doi:10.3389/fchem.2022.1094981)
Supplement: Supplementary file 1 [file DataSheet1.docx]

Supplementary Material

**
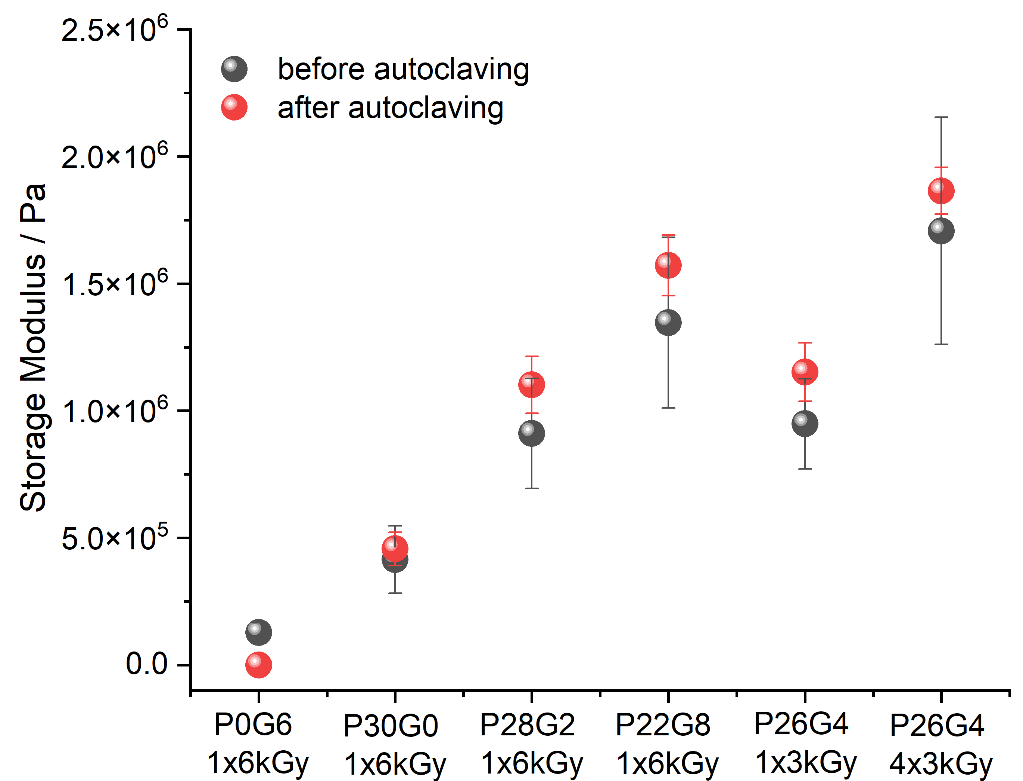
**

**Supplementary Figure 1.** Storage modulus P0G6, P30G0, P28G2, P22G8 irradiated with 6 kGy and P26G4 hydrogel irradiated with 1x3 kGy and 4x3 kGy. Moduli were measured at a frequency of 1 Hz. Error bars indicate the standard deviation.
